# Transcatheter aortic valve replacement in patients with a pre-existing prosthetic mitral valve: a single center experience with two cases

**DOI:** 10.1186/s43044-023-00433-7

**Published:** 2024-01-08

**Authors:** Oyewole A. Kushimo, Mitendra S. Yadav, Purneshwar Pandey, Santosh Singh, Viveka Kumar

**Affiliations:** 1 Max Institute of Heart and Vascular Sciences, Max Super-Specialty Hospital, Saket, New Delhi 110017 India; 2 Cardiology Unit, Department of Medicine, Lagos University Teaching Hospital, Lagos, Nigeria

**Keywords:** Transcatheter aortic valve replacement, Prosthetic mitral valve, Transfemoral, Case report

## Abstract

**Background:**

The performance of transcatheter aortic valve replacement (TAVR) in patients with pre-existing prosthetic mitral valves is technically challenging due to the potential interference between both prosthetic devices. At present, there are no clear recommendations for this patient subset due to their exclusion from clinical trials. We report our experience of two cases with pre-existing prosthetic mechanical mitral valves who underwent TAVR.

**Case presentation:**

The first case was a 57 year old man with severe aortic stenosis and type 2 diabetes mellitus who had a mitral valve replacement 32 years ago. Operative mortality risk assessed by the Society for Thoracic Surgery (STS) Score was 1.7%, but he was considered high risk in view of previous cardiac surgery. Pre-procedure CT evaluation revealed favorable aortic root and femoral access anatomy with the mechanical mitral valve located 6.3 mm below the aortic annular plane. He underwent TAVR with a Medtronic Evolut R 29 mm self-expanding transcatheter heart valve via the femoral approach.

The second case was a 66 year old lady who presented with severe aortic stenosis, atrial fibrillation and a history of mitral valve replacement 17 years ago for rheumatic mitral stenosis. Her STS score was 3.5%. Pre-procedure CT showed favorable aortic root and femoral access parameters with a mitral-aortic distance of 3.6 mm. TAVR was performed with a balloon expandable Myval 21.5 mm transcatheter heart valve via a transfemoral access. Both procedures were done successfully.

**Conclusion:**

This report highlights the feasibility of TAVR in post-mitral valve replacement patients provided careful pre-procedural evaluation, and planning is done.

**Supplementary Information:**

The online version contains supplementary material available at 10.1186/s43044-023-00433-7.

## Background

The development of transcatheter aortic valve replacement (TAVR) over the past two decades has led to a revolution in the management of valvular heart disease. It was initially indicated for high-risk patients with severe aortic stenosis (AS) unsuitable for surgical aortic valve replacement (SAVR). However, its use is expanding to patients with lower surgical risk, degenerated surgical bio-prosthesis and pure aortic regurgitation.

Patients who have undergone previous mitral valve replacement (MVR) represent a subset that has largely been excluded from trials demonstrating clinical and survival benefit of TAVR [1–3]. Existing data on the post-MVR TAVR procedure are limited to case reports/series [4–7] and retrospective analyses [8–11]. This explains why current guidelines have no specific recommendations for this subpopulation.

As a result of the close proximity of the aortic annulus and a non-compliant prosthetic mitral valve ring, performance of TAVR in post-MVR patients poses a technical challenge and the associated risk of interference between both prosthetic devices. Other potential risks include damage to prosthetic mitral leaflets by guide wires, higher probability of endocarditis and bleeding complications. We hereby report our experience with two patients with pre-existing mechanical mitral valve prosthesis who underwent TAVR with a self-expandable and a balloon expandable valve, respectively, via the transfemoral approach.

## Case presentation

### Case 1

**Fig. 1:**
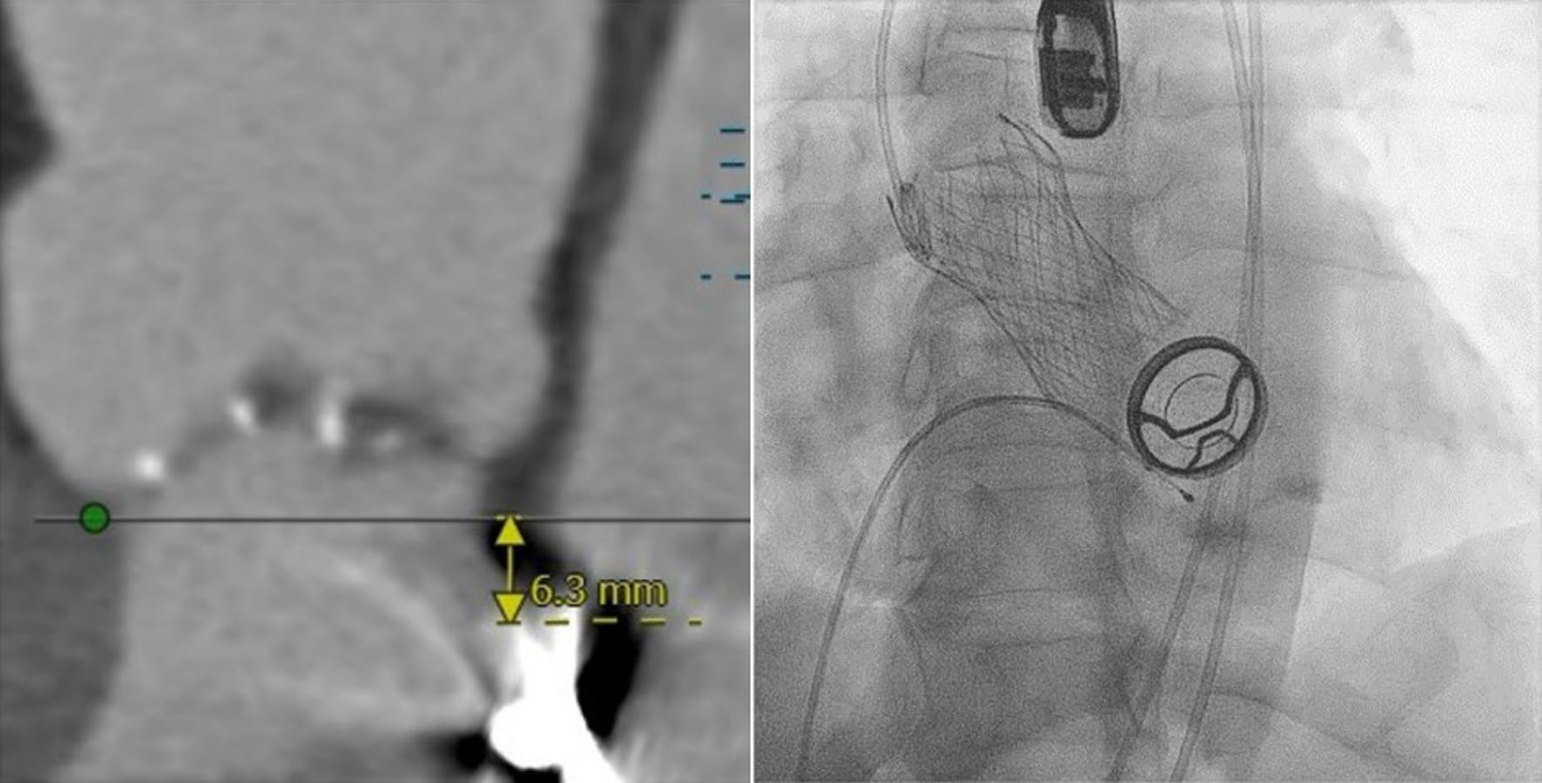
(case 1): On the left, pre-procedure Cardiac CT scan showing the prosthetic mitral valve—aortic annular distance. On the right, post-procedure fluoroscopic image showing deployed self-expandable THV in the aortic position and a mechanical PHV in the mitral position

A 57 year old man presented at our center with a 6months history of worsening breathlessness on exertion (NYHA class III) and background type 2 diabetes mellitus. He had a mitral valve replacement 32 years ago with a mechanical prosthetic valve. Echocardiography done revealed severe aortic stenosis with a mean gradient of 48 mmHg, normal functioning mechanical prosthetic mitral valve and left ventricular ejection fraction (LVEF) of 50%. Coronary angiography done was normal. Operative mortality risk assessed by the STS was 1.7%, but he was considered to be at high risk in view of previous cardiac surgery. He was scheduled for TAVR. Pre-procedure CT evaluation revealed favorable aortic root and femoral access anatomy with the mechanical mitral valve located 6.3 mm below the aortic basal annular plane (Fig. 1).

The TAVR procedure was done under moderate sedation with fluoroscopy and Trans-esophageal echo (TEE) guidance. Native aortic valve was crossed with a straight tip Terumo wire and Amplatz left 1 catheter which was subsequently exchanged with a pigtail catheter. A Confida wire was placed in the left ventricle over the pigtail. A self-expanding Medtronic Evolut R 29 mm Transcatheter heart valve (THV) with delivery catheter and 14F enline sheath was deployed (Fig. 1) under fluoroscopic and TEE guidance with RV pacing at 120 bpm (Additional file 1: Video S1, Additional file 2: Video S2). Post-deployment vitals were normal. Trans-aortic gradient by TEE was 9 mmHg. The patient discharged home after 5 days in a stable condition.

### Case 2

**Fig. 2:**
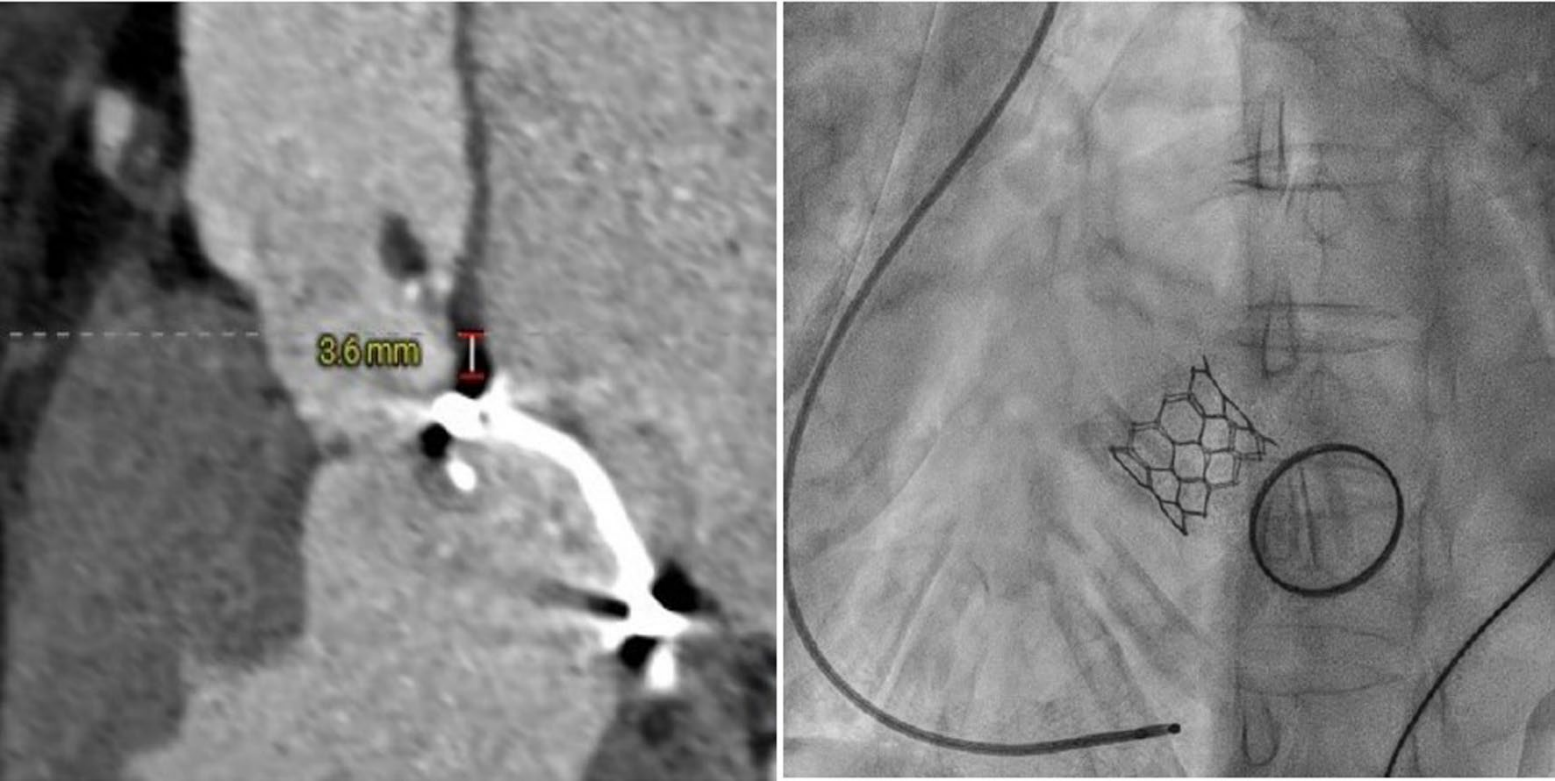
(case 2): On the left, pre-procedure Cardiac CT scan showing the prosthetic mitral valve—aortic annular distance. On the right, post-procedure fluoroscopic image showing deployed balloon expandable THV in the aortic position and a mechanical PHV in the mitral position

A 66 year old lady presented with a 5-day history of left sided chest pain. She had no history of hypertension or diabetes. Mitral valve replacement surgery with a mechanical prosthetic valve (27 mm, St. Jude) was done 23 years ago on account of severe mitral stenosis diagnosed 17 years earlier. Her ECG showed atrial fibrillation with slow ventricular response. Echocardiogram revealed severe aortic stenosis with a mean gradient of 74 mmHg and LVEF of 50%. CT coronary angiography showed non-obstructive CAD with a calcium score of 82.5. Her STS score was 3.5%. Pre-procedure CT showed favorable aortic root and femoral access parameters. Mitral valve—aortic annular distance was 3.6 mm (Fig. 2).

The TAVR procedure was done under moderate sedation with fluoroscopy guidance. Native aortic valve was crossed as described above. A Safari wire was placed in the left ventricle over a pigtail catheter. A balloon expandable pre-loaded Myval 21.5 mm THV (Meril life sciences, Gujarat, India) with delivery system was inserted under fluoroscopic guidance. The valve was deployed (Fig. 2) in the aortic position during rapid RV pacing at 180 bpm (Additional file 3: Video S3). Aortic root angiography confirmed proper positioning, no significant regurgitation and good flow in the coronary arteries (Additional file 4: Video S4). Post-deployment trans-aortic gradient decreased to 5 mmHg assessed by transthoracic echo.

Patient developed complete heart block a few hours post-procedure for which she had a single chamber permanent pacemaker inserted the next day. She was discharged home after 4 days in a clinically stable condition.

## Discussion

About three quarters of patients who undergo mitral valve surgery develop aortic valve disease during follow-up, with 5% of them requiring a new intervention [12]. Prior cardiac surgery in SAVR candidates increases operative risk by up to 70% [13]. Furthermore, the presence of a prosthetic mitral valve (PMV) doubles the mortality in patients undergoing SAVR compared with those without a pre-existing PMV [14]. For these reasons, TAVR seems to be a reasonable alternative for this high-risk population in view of its extensive use and proven efficacy in high- and intermediate-risk patients with severe aortic stenosis [1–3]. Our experience with the two reported index cases, similar to other reports and registry data included in a recent systematic review [15], suggests the feasibility of this course of action. Furthermore, it seems the presence of a PMV may not significantly increase short term mortality risk post-TAVR [9, 11]. However, randomized clinical trial data and guideline recommendations are still lacking.

In view of the technical concerns and risk of interference between the implanted THV and the pre-existing PMV, careful pre-procedural planning with CT and TEE imaging is highly recommended. Interference between the THV and PMV may lead to acute/chronic malfunction of both prostheses and an increased risk of device embolization [15]. The latter was noted among 6.7% of post-MVR patients in a multicenter Spanish registry [9]; however, this was not significantly higher compared with patients without prior mitral valve surgery. Furthermore, all cases of embolization occurred in subjects with the PMV to aortic annulus distance of < 7 mm by CT imaging. A minimum mitral-aortic distance of 3 mm for the balloon expandable Sapien valve and 4 mm for the self-expandable CoreValve has been recommended [8]. The two index cases had a distance of 6.3 mm and 3.6 mm, respectively, which are within the acceptable minimum range.

The preferential use of self-expandable valves in post-MVR TAVR procedures may seem logical in view of the possibility of partially recapturing the prostheses during deployment. However, recent reports and retrospective analyses have demonstrated similar peri-procedural outcomes with the use of either self or balloon expandable valves [8, 9, 15].

Bleeding complications have been reported to be more frequent in subjects with PMV who undergo TAVR [9]. This is likely due to the need for more aggressive antithrombotic therapy because of a higher rate of atrial fibrillation and the presence of a mechanical PMV. We did not observe any bleeding complication among the 2 index cases.

Our second patient developed complete heart block a few hours post-TAVR. The peri-procedural occurrence of conduction abnormalities is reportedly the most common complication of TAVR with about 13% of subjects requiring permanent pacemaker implantation [16]. This is due to the close proximity of the aortic valve apparatus to the AV node, HIS bundle and the proximal left bundle branch. This explains the high peri-procedural incidence AV block and new onset left bundle branch block [16]. Factors associated with the occurrence of these abnormalities include prior right bundle branch block, transcatheter valve type and implantation depth [16]. Permanent pacemaker implantation is about 5 times more frequent among subjects receiving self-expandable CoreValve (25–28%) compared with subjects with a balloon expandable Edwards SAPIEN/SAPIEN XT valve (5–7%). This observation is likely related to the deeper implantation required for self-expandable valves [16]. It is not clear whether post-MVR subjects are at a higher risk of post-TAVR conduction abnormalities. However, our second case likely had an AV conduction disturbance in view of her prior history atrial fibrillation with slow ventricular response. This might have increased her risk of developing complete heart block post-procedure.

## Conclusions

The performance of TAVR in subjects with pre-existing prosthetic mitral valves seems feasible and safe provided there is thorough pre-procedure evaluation and planning. Inclusion of these patients in clinical trials may provide further insights and a basis for specific recommendations in the future.

### Supplementary Information

**Additional file 1: Video S1**. Video shows the deployment of a self-expandable THV in the aortic position. (Case 1)

**Additional file 2: Video S2**. Video shows the aortic root angiography confirming optimal deployment of the THV. (Case 1)

**Additional file 3: Video S3**. Video shows the deployment of a balloon expandable THV in the aortic position. (Case 2)

**Additional file 4: Video S4**. Video shows the aortic root angiography confirming optimal deployment of the THV. (Case 2)

## Data Availability

Not applicable.

## Abbreviations

TAVR: Transcatheter aortic valve replacement
AS: Aortic stenosis
SAVR: Surgical aortic valve replacement
MVR: Mitral valve replacement
LVEF: Left ventricular ejection fraction
TEE: Transesophageal echocardiography
THV: Transcatheter heart valve
PMV: Prosthetic mitral valve
